# Study on ASJ Cutting of TC18, Based upon Multivariate Nonlinear Regression and SA-BP-AGA

**DOI:** 10.3390/ma12121902

**Published:** 2019-06-13

**Authors:** Jie Lin, Xin Zhou, Hui Zhang, Fengchao Wang, Qiwen Xu, Chuwen Guo

**Affiliations:** 1 School of Electrical and Power Engineering, China University of Mining and Technology, Xuzhou 221116, China; TS17130003A3@cumt.edu.cn (J.L.); jianniang_zhouxin@163.com (X.Z.); cumthui@126.com (H.Z.); wfc0317@163.com (F.W.); xqw0703@163.com (Q.X.); 2 Xuhai College, China University of Mining and Technology, Xuzhou 221116, China

**Keywords:** ASJ cutting, retardation coefficient, SA-BP-AGA, TC18

## Abstract

TC18 titanium alloy has been widely applied, but is considered as a difficult machining material. Taking the kerf angle as the quality criterion, this paper studied the cutting performance of TC18 by the use of an abrasive slurry jet (ASJ), based upon multivariate nonlinear regression and SA-BP-AGA. Cutting experiments were carried out according to the Taguchi orthogonal method. The experimental factors included traverse speed, standoff distance, pressure and slurry concentration, with five levels set, respectively. Meanwhile, a characterization method of the major influencing factors was proposed. A multiple nonlinear regression model and a back propagation artificial neural network (BP) prediction model, based on adaptive genetic algorithm (AGA), were established. The reliability was verified by statistics equations for the 22 groups of the fitting or training model and the three groups of experimental results. The BP-AGA and Simulated annealing algorithm (SA) were used to form a set of prediction optimization systems, called integrated SA-BP-AGA. Finally, the results showed that the main factor influencing the kerf angle is the slurry concentration. BP-AGA is easier to model, offers better robustness and is more accurate than a multivariate nonlinear regression model. The best kerf angle can be predicted by the integration system. The study results can improve the performance for the machining of TC18 by ASJ.

## 1. Introduction

Titanium alloy TC18 (Ti-5Al-5Mo-5V-1Cr-1Fe) possesses the common excellent performance of both alpha phase and beta phase titanium alloy, such as high strength to weight ratio, high toughness, high hardness, high corrosion resistance, being non-magnetic and so on, with a new style alloy (alpha + beta). It has been widely used in aviation, biomedical, automotive fields, etc. [1,2]. However, there exist enormous challenges for traditional machining approaches due to its properties, including poor heat transfer performance, work-hardening and unstable chemical reaction, and deformation under high temperature conditions, which tends to cause serious tool wear, even much lower durability and shorter life than expectancy [3,4,5]. Moreover, some researches showed that the quality of the machined surface of the work piece has significant influence on its mechanical properties, especially upon fatigue properties [6,7,8]. To address these issues, some researchers used an improved genetic algorithm to optimize the milling parameters of TC18, and studied the forging process parameters of TC18 based upon the BP neural network, but it did not overcome the negative effect of the poor property of TC18 on the quality of the traditional processing technology, such as the heat affected zone, etc. [9,10]. Therefore, there is an urgent need for an advanced processing method to solve various problems that arise in the processing of TC18.

ASJ technology is one of the fastest growing and most advanced non-traditional processing technologies. It has the advantages of no thermal effects, no residual stress, good incision quality, high applicability of materials, being environment friendly and highly competitive in material processing [11]. Wang studied the mechanism of kerf width and kerf angle formation during abrasive water jet machining [12]. Azmir used the Taguchi experimental method and variance analysis to study the influence of processing parameters on the kerf angle upon the cutting of glass/epoxy composite laminate, and concluded that the type of abrasive is the most important controlling factor [13]. Alberdi established a mathematical model based on pressure, the mass flow of the abrasive, target distance and transverse velocity processing parameters, which is used to predict the profile produced by AWJ cutting 1075-T651 [14]. Feng used numerical simulations and experiments, concluding that the jet with the added polymer has better stability in air [15]. Wang found that adding high polymer to the abrasive slurry to cut stainless steel would have better processing performance [16]. Just as traditional machining relies on computer optimization control and an optimization of processing efficiency [17,18,19], advanced computer algorithms can also be used to optimize the processing parameters of an abrasive water jet in order to obtain high-quality products. Azlan used an integrated system of SA and GA algorithms to optimize the parameters of the abrasive processing process [20]. However, it depends on a great multivariate nonlinear regression model which is difficult to obtain. By considering the diameter of the focused nozzle and controllable process parameters such as work pressure, traverse speed and abrasive flow rate, Srinivasu modeled the artificial neural network to predict the depth of cut in the AWJ process, and also used a genetic algorithm to find out the optimal parameters combination [21]. However, the accurate ANN (artificial neural networks) prediction modeling was constructed directly with enormous work and difficulty.

In order to study the effect of processing parameters on TC18, we used Taguchi’s orthogonal method to carry out the experiments. To optimize the machining process by ASJ for TC18, and at the same time taking into account the stability of the jet pressure in the experiment, we provided some new measures to analyze the experimental data. Firstly, a multivariate nonlinear regression model was established, and the reliability of the prediction model was verified by using a mathematical statistics formulae (MAPE, MSE and R^2^) and some specific experimental data. Based on the verification results, the model was only used to determine the main influencing factors of the experiment, which indicated that Azlan’s method [20] was not applicable here. We comprehensively utilized the good methods proposed by Azlan [20] and Srinivasu [21], meanwhile avoiding the restrictive conditions in [20] that it must rely on a great nonlinear prediction model, and solved the difficult problem of directly establishing the neural network prediction model in [21]. A back propagation artificial neural network (BP) prediction model, based on adaptive genetic algorithm (AGA) was established, and of which (BP-AGA) the validation was checked by using the same method as above. Then this study compared the multiple nonlinear regression method with the neural network method optimized by the adaptive genetic algorithm. It is found that BP-AGA is easier to model, offers better robustness and is more accurate. Finally, the BP-AGA and simulated annealing algorithm (SA) optimization technology were used to form a set of prediction systems, called integrated SA-BP-AGA. Through this integrated system, the best kerf angle and the parameters affecting the kerf angle were obtained. The study results can improve the performance for TC18 machining by ASJ.

## 2. Experiment

### 2.1. Experimental System

**Figure 1:**
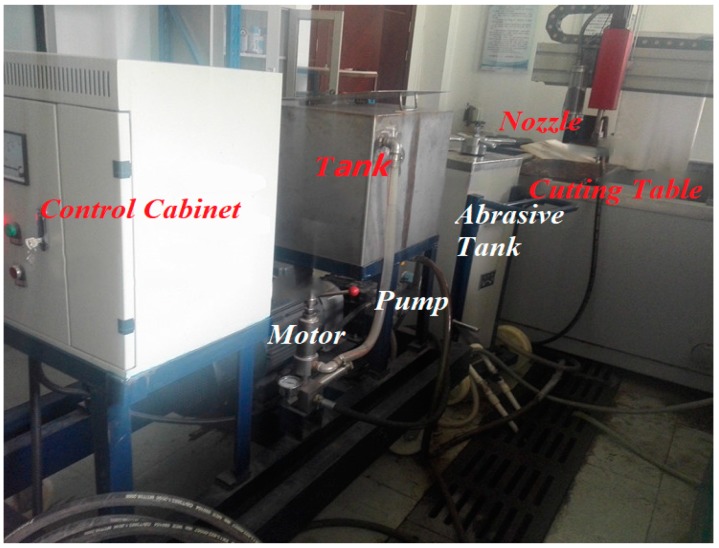
DWJ1313-FC Abrasive Jet Machining system.

The experiment was carried out on the DWJ1313-FC abrasive jet cutting system (DARDI, Nanjing, China) at the Water Jet Research Center of the China University of Mining and Technology, and the set of equipment is shown in Figure 1.

As shown in Figure 1, the experimental equipment consists of a control cabinet, CNC (computerized numerical control) cutting table, booster pump, the water tank, an abrasive tank, etc. Firstly, the working pressure is set by the control cabinet, and then the booster pump is started to force the polyacrylamide (PAM) slurry in the water tank flow into the abrasive tank at high speed, and then mix with the abrasive particles to form the abrasive slurry high-speed fluid. Then the steady jet is polymerized by the jet nozzle on the numerical control cutting platform. Finally, the cutting platform can be activated, and the nozzle will carry out the cutting experiment according to the planning road path.

### 2.2. Experimental Scheme

**Figure 2:**
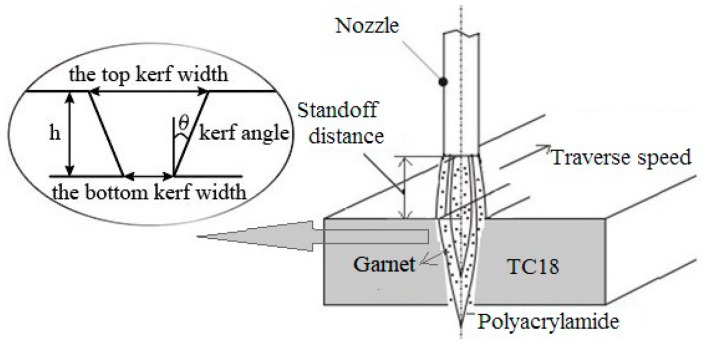
Processing principle schematic.

As shown in Figure 2, there are many processing parameters to be set in the ASJ cutting experiment. In this experiment, the effects of traverse speed, standoff distance, system pressure and slurry concentration on the cutting quality characteristics (the kerf angle), are studied. The kerf angle indicates the inclination of the cutting wall. To solve the problems of expensive costs and inefficiency caused by the full-factor experimental method, we adopted the Taguchi orthogonal theory to carry out experiments [22]. Five levels were set for each variable, as shown in Table 1, and other processing parameters were kept constant for all cuts, as shown in Table 2. Based on the same experimental system, Wang [12] provided a reference for the experimental processing parameter design of this paper.

### 2.3. Experimental Results

**Figure 3:**
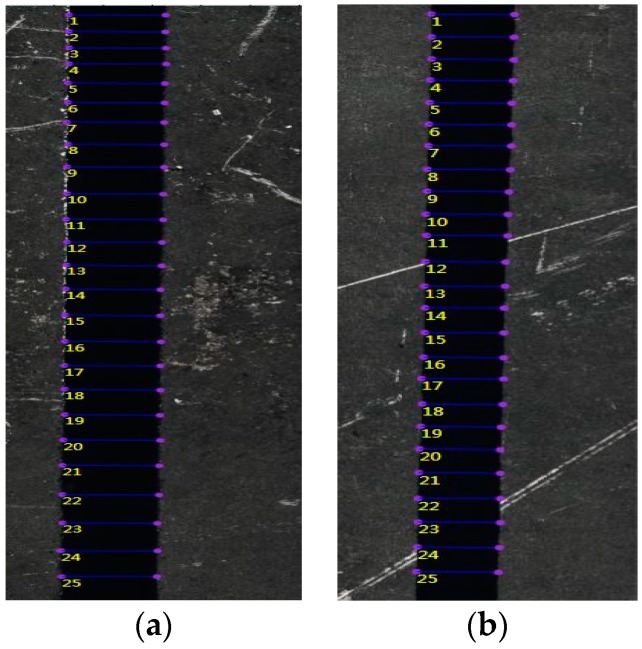
Measure mode diagram. (**a**) Top kerf width; (**b**) bottom kerf width.

This experiment followed the L25 (5^4^) orthogonal experiment table designed by MINITAB 17 software. The actual processed data and the measured characterization data were shown in Table 3. The kerf width was measured with an OLYMPUS DSX510 Microscope (Olympus, Tokyo, Japan). For high-quality image acquisition, we used an external 10× objective lens and an internal default 50× eyepiece combination. Then, through the DSX software system, we set the focal length to 1×, then set the acquisition mode to a 3D bright field, and set the image stitching overlap ratio to 20%, and finally set the acquisition area to 10 mm × 3 mm. After the automatic acquisition and splicing were completed, the geometric measurement mode in the DSX510 software measurement module was selected to measure the kerf width in the high quality image. In order to reduce the error caused by the measurement, the top kerf width and the bottom kerf width corresponding areas of the same cut sample, were respectively measured 25 times, and the average values were taken as their final measurement results. Image measurement is shown in Figure 3.

The inclination of the kerf is defined as:

(1) $$q=\tan^{-1}\left(\frac{W_{\mathrm{top}}-W_{\mathrm{bottom}}}{2h}\right)$$

where, θ, W_top_, W_bottom_, and h are the kerf angle, the thickness of the cutting specimen, the top kerf width, and the bottom kerf width, respectively, as shown in Figure 2.

## 3. Predictive Model of Kerf Angle based on Multivariate Nonlinear Regression Modeling

### 3.1. Methodology

Due to the slight fluctuation of the instability of the jet pressure during the real processing, the fluctuation error can be quantized by the mean relative level error function (MRLE).

(2) $$\mathrm{MRLE}=\frac{1}{d}\sum_{i}\left(\frac{\left|t_{i}-o_{i}\right|}{n}\right)\times 100\%$$

where, d is the tolerance among the adjacent levels, t_i_ is the actual experiment value of the group i, o_i_ is the designed value or the predictive value, n is the number of designs at the same level.

**Figure 4:**
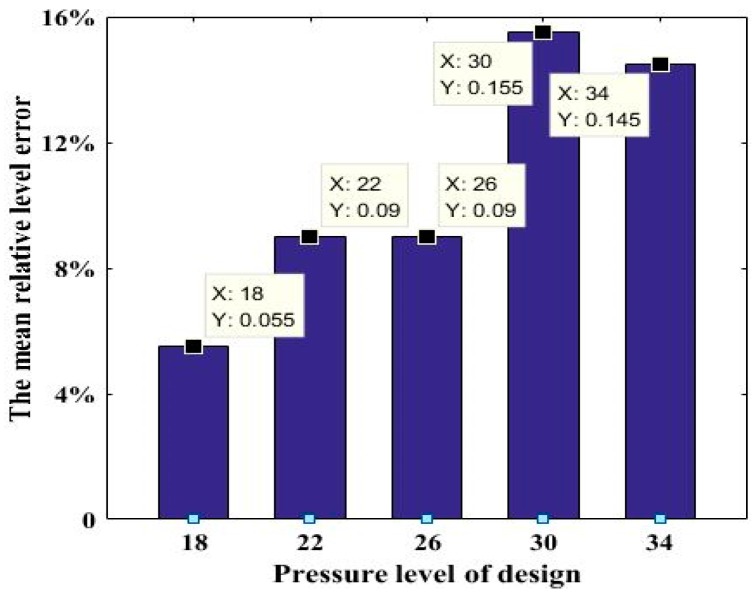
Mean relative pressure level error.

The Mean relative pressure level error calculated by the above Equation (2) is shown in Figure 4. In Figure 4, the corresponding value of Y is the decimal form of MRLE. It can be seen from the diagram that the maximum average relative error is 15.5%. Therefore, the commonly used analysis method-single factor multivariate variance based upon the Taguchi orthogonal method cannot be applied in this article.

Therefore, we proposed a new idea to deal with this kind of situation. First of all, through multivariate nonlinear regression methods, the fitting was performed on the actual jet pressure values in all the odd groups and all the even numbers except (18, 22, 24) in Table 3. Then, the fitting degree of the regression equation was determined based on the multivariate correlation coefficient R, after which the reliability of the prediction model was verified by mathematical statistics formulas with the 22 groups’ modeling data and the remaining three groups’ data of experiments. These formulae included the determination coefficient (R^2^), mean squared error (MSE) and the mean absolution percentage error (MAPE).

(3) $$R=\frac{\sum (t_{i}-\bar{t})\sum \left(o_{i}-\bar{t}\right)}{\sqrt{\sum {\left(t_{i}-\bar{t}\right)}^{2}\sum {\left(o_{i}-\bar{t}\right)}^{2}}}$$

(4) $$R^{2}=1-\left(\frac{\sum_{i}{\left(t_{i}-o_{i}\right)}^{2}}{\sum_{i}{\left(o_{i}\right)}^{2}}\right)$$

(5) $$\mathrm{MSE}=(\frac{1}{N}{\sum_{i}\left|t_{i}-o_{i}\right|}^{2})$$

(6) $$\mathrm{MAPE}=(\frac{1}{N}\sum_{i}\left|\frac{t_{i}-o_{i}}{t_{i}}\right|\times 100)$$

where t is the average of the actual experiment value; N is the total amount of participating in the calculation of runs.

Finally, partial derivatives of each processing variable of the established regression equation were calculated. Substituting the values of the processing variables in each group into the partial derivative equation, the slope of the aimed partial variable of the regression equation in each group coordinates was obtained, as described in Equation (7).

(7) $${\mathrm{Slope}}_{\left(k\right)}=f_{k}^{\prime }(V,H,C,P){|}_{\left(V_{i},H_{i},C_{i},P_{i}\right)}$$

where, Slope_(k)_ is the slope of the equation about the k variable, k can be V, H, C or P. In addition, V_i_, H_i_, C_i_ and P_i_ are the values of group i in Table 3, respectively.

These comprehensive values of the slope of each variable can be figured out to characterize the sensitivity of the kerf angle to each processing variable, which are shown as the Equations (8)–(11).

(8) $$\mathrm{Mean}(S_{k})=\frac{1}{n}\sum_{i}^{n}Slope_{(k)i}$$

(9) $$\mathrm{Ct}=\Delta \theta /\left(\frac{\Delta k}{k_{max}-k_{min}}\right)=\frac{\Delta \theta }{\Delta k}\times (k_{max}-k_{min})$$

In which,

(10) $$\frac{\Delta \theta }{\Delta k}=\mathrm{Mean}(S_{k})$$

where, Mean (S_k_) is the mean of each variable’s slope; Ct means that when the other variables are unchanged, the change of k relative to its own interval will theoretically cause the maximum change of θ; and k represents one of the variables selected in the experiments.

(11) $$\mathrm{Cr}=\left|\frac{\theta_{\max}-\theta_{\min}}{\mathrm{Ct}}\right|$$

where, Cr is the interval length of the actual kerf angle change divided by the Ct (in theory, the maximum change length of the k variable can cause the maximum change in the kerf angle). This also means that this k factor hinders the change of inclination caused by the change of the other factors through its own changes, which is called the retardation coefficient of k. 

The smaller the retardation coefficient is, the more powerful the influence of k is. The method was compared with the results of multiple variances, and it was found that when the Cr corresponding to the factor was less than 10, the factor had an important influence.

Through this method, the influence of factors can be sorted, and the main influencing factors are identified.

### 3.2. The Regression Model of Kerf Angle 

What is given here mainly showed the transformation of the original data by us to complete the regression fitting. The mathematical model established in this paper is expressed as Equation (12), which is one of the commonly used fitting basic equations in the field of waterjet machining [20,23,24].

(12) $$\theta ={\mathrm{aV}}^{q}H^{s}{\left(C+C_{0}\right)}^{z}P^{u}e^{\prime }$$

where, ε’ is the experimental error, and a, q, s, C_0_, z and u are pending parameters to be estimated by the experimental data.

Equation (12) can be linearized by performing a logarithmic transformation as follows:

(13) $$\ln\theta =\mathrm{lnc}+qlnV+slnH+tln(C+C_{0})+ulnP+lne^{\prime }$$

The final Equation (13) can be written as:

(14) $$\hat{\theta }={\mathrm{aV}}^{q}H^{s}{\left(C+C_{0}\right)}^{t}P^{u}$$

The regression model for the kerf angle that has been determined, is as follows:

(15) $$\hat{\theta }=2.038V^{0.0094}H^{-0.0088}{\left(C+1\times 10^{-5}\right)}^{-0.0971}P^{-0.0896}$$

The multivariate correlation coefficient R is calculated as the linearized regression Equation (13), and R = 0.8316, greater than 0.8, which can be accepted. The rest of the mathematical statistics test is calculated according to Equation (15). Using the remaining 3 groups of experimental parameters to check the prediction accuracy of the equation, and quantified by statistical formulae R^2^, MSE, and MAPE. The results listed into the table 5 are 0.8383, 0.6415, and 69.4384%, respectively. Although the mean absolute error percentage exceeds 20%, indicating that the regression model prediction accuracy is not high, the coefficient of determination and the average error are both within acceptable limits, and the statistical result of training groups are 0.9558, 0.3647, and 19.5027, which are listed into the Table 4. So the training groups can be used to assess the influence of the four factors.

### 3.3. Analysis of Main Influencing Factors

**Figure 5:**
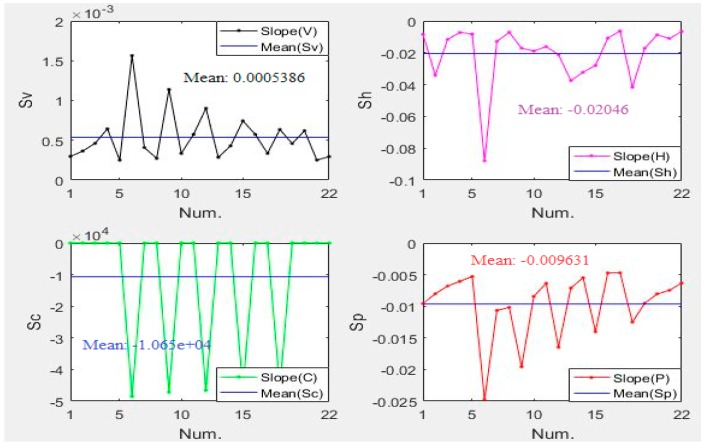
Slope (V, H, C, P).

By Equations (7) and (8), the slope of each variable is calculated and expressed in Figure 5.

As shown in Figure 5, it can be obviously observed that the slope fluctuation of the slurry concentration is particularly intense, and its absolute mean value is also large, which is several orders of magnitude larger than the other three variables. So a simple estimate can be obtained that the main influencing factor is C. By Equation (11), the Cr values of V, H, C and P are 228.77, 116.5388, 0.0025, and 32.61, respectively. It can be clearly known that C has a significant influence, followed by P, H, and V, which has little influence.

## 4. Predictive Model of Kerf Angle Based on ANN-AGA

It can be seen from the above calculation results that the multivariate nonlinear regression is not good at prediction. Therefore, a new prediction model is established.

### 4.1. Methodology

During neural network training, network structure parameters and initial thresholds and weights determine the training duration and network quality of the network to a large extent. Due to the nature of the “black box” of neural networks, it leads to blindness in debugging and low training efficiency. 

However, by using the AGA method to find the optimal initial training thresholds and weights of the neural network, the blindness of debugging is reduced to a certain extent, the efficiency of network training is greatly improved, and the quality of the network is indirectly improved. Finally, the test results are analyzed by the same statistical formulae (R^2^, MSE and MAPE).

**Figure 6:**
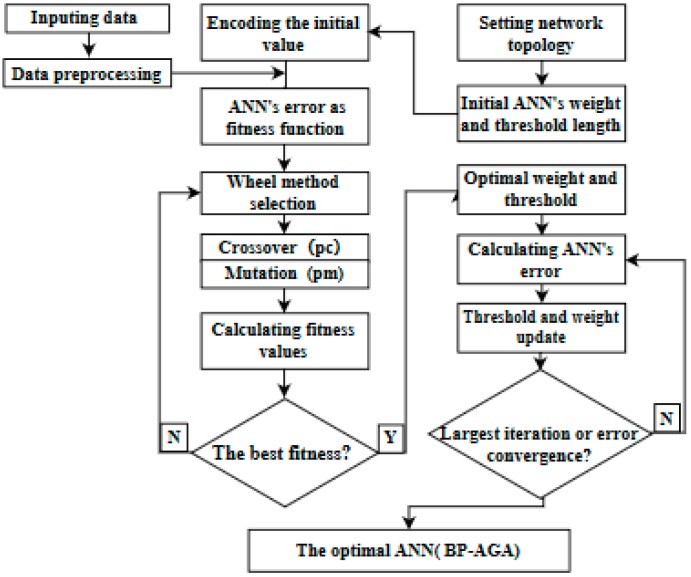
Back propagation-adaptive genetic algorithm (BP-AGA) structure schematic diagram.

An adaptive genetic algorithm optimizes the BP neural network flow as shown in Figure 6.

After debugging the structure of neural network and random initial weights and thresholds, it is supplemented by the adaptive genetic algorithm to improve efficiency and quality. This genetic algorithm mainly includes chromosome coding, selection operation, mutation operation, crossover operation and fitness operation. Among them, the crossover ratio (pc) and the mutation ratio (pm) in the parameters of the genetic algorithm play very important roles in the performance of the algorithm. If the fixed pc and pm values are adopted, it is difficult to adapt to the change of population, and sometimes leads to the evolution of the past. In this paper, an adaptive algorithm based on Srinvivas is proposed. The pc and pm in the algorithm can change automatically with fitness values, which can maintain group diversity and ensure convergence, as shown in Equation (16) below.

(16) $$\begin{array}{l} \mathrm{pc}=\{\begin{array}{ll} {\mathrm{pc}}_{\max}-\frac{\left({\mathrm{pc}}_{\max}-{\mathrm{pc}}_{\min})(f_{\max}-f^{\prime }\right)}{f_{\max}-f_{avg}}, & f^{\prime }\geq f_{avg}\\ {\mathrm{pc}}_{\max}, & f^{\prime }<f_{avg} \end{array}\\ \mathrm{pm}=\{\begin{array}{ll} {\mathrm{pm}}_{max}-\frac{\left(pm_{max}-pm_{min})(f_{max}-f\right)}{f_{max}-f_{avg}}, & f\geq f_{avg}\\ {\mathrm{pm}}_{max}, & f<f_{avg} \end{array} \end{array}$$

where, pc_max_, pc_min_, pm_max_ and pm_min_ are the maximum, minimum crossover rate and mutation rate, respectively. f_max_, f_avg_ are the maximum fitness value and the average fitness for each generation of population. f’ is the larger fitness value of the two individuals to cross, and f is the fitness value of the variant individuals.

Firstly, the chromosome was constructed by a binary encoding of the initial threshold and weight of the neural network, and the prediction error of the neural network was used as our fitness value. Then the individual difference was produced by chromosomal variation and cross, and the selection of the wheel was executed by the principle of survival of the fittest. 

The optimal threshold and weight of the neural network were found. Finally, based on these parameters, the neural network was trained to get the best network model.

### 4.2. Neural Network Optimized by Adaptive Genetic Algorithm Based on Kerf Angle

**Figure 7:**
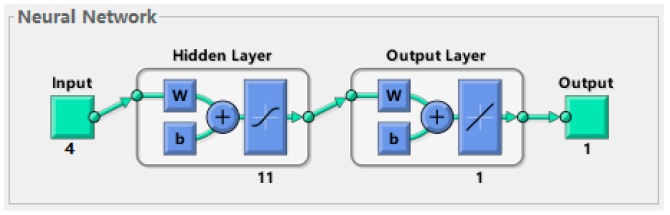
ANN’s (BP-AGA) structure diagram.

**Figure 8:**
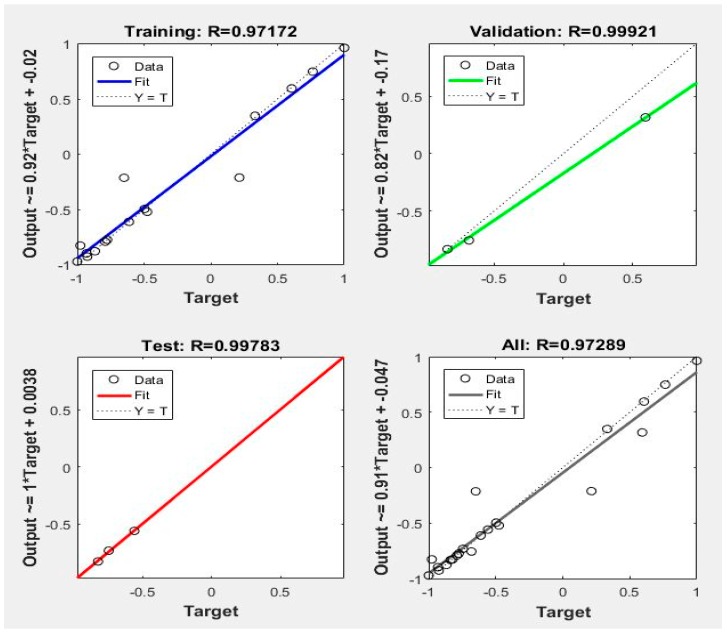
ANN’S (BP-AGA) regression performance diagram.

It was finally determined that the structure of ANN is 4-11-1, that is, with 4 input nodes, 11 hidden nodes and 1 output nodes. The adaptive genetic algorithm parameters included, the population size is 24, the maximum cross rate is 0.7, the minimum cross rate is 0.1, the maximum mutation rate is 0.05, and the minimum mutation rate is 0.01. Similarly, the 22 sets of data selected above were used for training. A neural network prediction model with high accuracy was established by MATLAB 2016. The structure of the neural network is shown in Figure 7. The regression performance of the neural network is shown in Figure 8. 

From Figure 8, it can be found that the multivariate correlation coefficient R of training and testing is 0.97172 and 0.99783, respectively, with a high goodness of fit, listed in the Table 4. Then, the remaining three groups are still used as checking groups, which are calculated by the statistical formulae R^2^, MSE and MAPE. The results are listed into the Table 5.

## 5. Comparison of the Two Analysis Methods

Currently, the main common methods for multivariate regression analysis are Forward, Forward, Backward and Stepwise. But in most cases, they are difficult to fit and are prone to multiple collinear troubles [24]. Therefore, a large amount of time has to be used to perform various transformations on the data to obtain a higher coefficient of multiple correlation and determination. 

However, using the artificial neural network to learn experimental data, and then predicting, only the optimization algorithm is needed to optimize the initial threshold and weight of the network, and then the simple network structure parameter adjustment can achieve the purpose.

From Table 4 and Table 5, the fitting quality and prediction performance of the regression model are not as good as the training quality and prediction performance of neural network model. Moreover, compared with the statistical analysis results of training quality and prediction performance, it can be seen that the neural network has better robustness and fault tolerance than the fitting regression method.

## 6. The Integrated SA-BP-AGA Optimization

**Figure 9:**
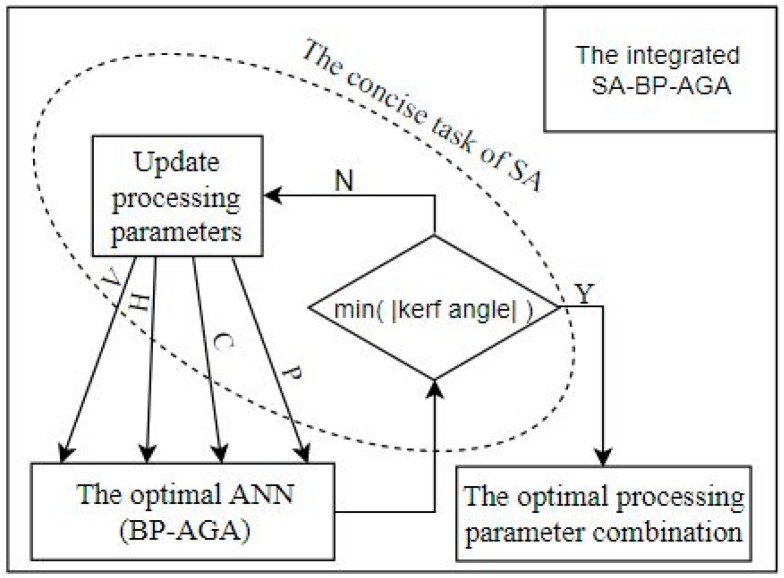
SA-BP-AGA structure schematic diagram.

Based on the trained neural network prediction model, a simulated annealing algorithm was used to find the optimal parameter combination of the minimum processing kerf angle. The integrated SA-BP-AGA of the above description is shown in Figure 9.

**Figure 10:**
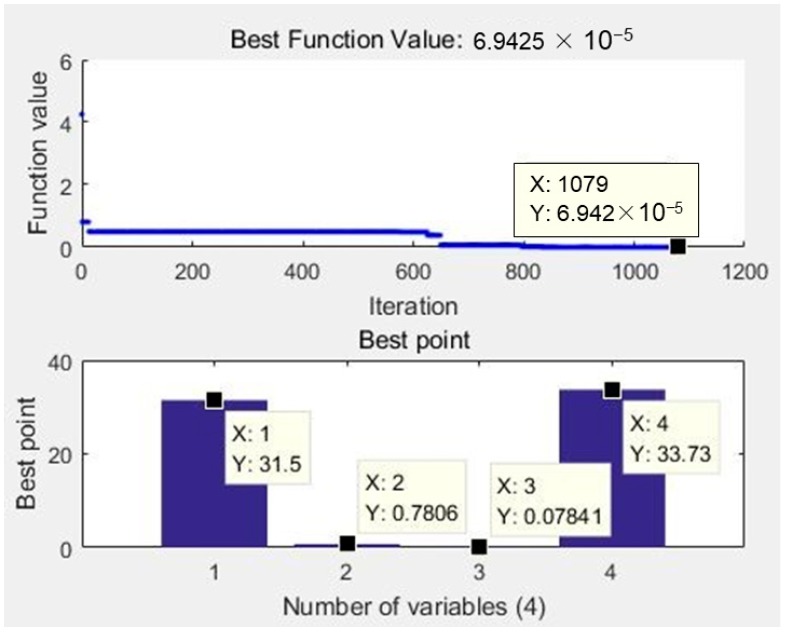
Fitness function plot of SA-BP-AGA.

The simulated annealing algorithm is a random search technique that is able to escape local optima using a probability function [25]. SA is a relatively mature algorithm, widely used in VLSI (Very Large Scale Integration) design, image recognition and neural network computer research. It can be decomposed into three parts: Solution space, objective function and initial solution. Here, the solution space is composed of the ranges of various processing variables. The objective function is the AGA-optimized neural network, and the initial solution is set as the best parameter group that appears in the experiment, which is the 24th in the Table 3. The optimal solutions of the MATLAB Optimization Toolbox is given in Figure 10 on the base of these criteria as listed in Table 6.

As shown in Figure 10, the theoretically optimal solution was observed that the minimum kerf angle is 6.9425 × 10^−5^. The set value of process parameters that lead to the minimum θ value are 31.5 mm/min for traverse speed, 0.7806 mm for standoff distance, 0.07841% for slurry concentration and 33.73 MPa for jet pressure.

## 7. Conclusions

The paper carried out experiments according to Taguchi’s orthogonal method, and established two predictive models for kerf taper based on a multivariate nonlinear model and the BP-AGA model. Meanwhile, statistical formulae (MAPE, MSE and R^2^) were used to verify and compare the reliability of the two prediction models. The BP-AGA prediction model is more reliable than the multivariate nonlinear regression prediction model. Moreover, neural network prediction shows better robustness and fault tolerance. The main influencing factors can be determined by the retardation coefficient proposed in this paper. This coefficient proposes to broaden the application of the Taguchi orthogonal test method in the field of the non-precision control industry, and provides a solution for the study of fluctuation factors by the Taguchi orthogonal horizontal experiment method. It was found that the main factor affecting the kerf angle is slurry concentration, and then the system pressure and standoff distance, while the traverse speed has barely influence. The optimal processing parameter combination under the condition of the minimal kerf angle are obtained by SA based on the prediction model of BP-AGA. The study results can improve the performance for TC18 machining by ASJ and guide actual production.

## Figures and Tables

**Table 1:** Variable parameters and their levels.

Number	Variables	L1	L2	L3	L4	L5
1	Traverse speed V (mm/min)	30	40	50	60	70
2	Standoff distance H (mm)	0.5	1	1.5	2	2.5
3	Slurry concentration C (%)	0	0.05	0.1	0.15	0.2
4	System pressure P (MPa)	18	22	26	30	34

**Table 2:** Constant Parameters.

Invariables	Values
Material size	200 × 30 × 5 (mm^3^)
Nozzle diameter	1.0 (mm)
Volume fraction of abrasive	20%
High Polymer	PAM
Average diameter of abrasive	0.27 (mm) or 80 (mesh)
Abrasive material type	garnet
Angle of influence	0 (degree)

**Table 3:** The experimental data for model constructions.

NO.	Operating Variables					Result		
	V	H	C	P	Actual P	Top Kerf Width (mm)	Bottom Kerf Width (mm)	Kerf Angle (°)
1	1	1	1	1	18.1	0.880234	0.51703	4.1547
2	2	2	4		18.4	0.901138	0.752207	1.7061
3	3	3	2		18.3	0.940592	0.820456	1.3764
4	4	4	5		17.8	0.896659	0.708886	2.1507
5	5	5	3		17.9	1.013852	0.867836	1.6727
6	1	4	3	2	22.1	1.027128	0.852151	2.0043
7	2	5	1		22.2	1.003986	0.516018	5.574
8	3	1	4		21.7	0.913358	0.732508	2.0715
9	4	2	2		22.8	0.909006	0.77915	1.4877
10	5	3	5		22.4	0.908018	0.767484	1.61
11	1	2	5	3	25.9	0.901708	0.785861	1.3273
12	2	3	3		25.9	0.940733	0.59944	3.9049
13	3	4	1		26.1	0.958826	0.514941	5.0732
14	4	5	4		26.7	0.999784	0.786822	2.4389
15	5	1	2		26.8	0.925032	0.767567	1.8038
16	1	5	2	4	30.5	1.048323	0.919693	1.4737
17	2	1	5		30	0.923354	0.768629	1.7725
18	3	2	3		30.6	0.979514	0.822616	1.7973
19	4	3	1		30.4	0.946768	0.532342	4.7381
20	5	4	4		31.6	0.996658	0.798935	2.2646
21	1	3	4	5	34.8	1.027287	0.864021	1.8702
22	2	4	2		33.5	0.965137	0.852903	1.2859
23	3	5	5		34.1	0.991862	0.782701	2.3954
24	4	1	3		35.4	0.927152	0.855971	0.8156
25	5	2	1		33.9	0.960782	0.54892	4.709

**Table 4:** Fitting or Training quality performance.

Model	Fitting or Training Quality		
	MSE	MAPE (%)	R^2^
Regression	0.3647	19.5027	0.9558
network	0.0987	6.0343	0.9881

**Table 5:** Prediction quality.

Model	Number			Prediction Quality		
	18	22	24	MSE	MAPE (%)	R^2^
Regression	1.9460	2.0897	1.9359	0.6415	69.4384	0.8383
network	1.8161	1.2147	0.8946	0.0039	5.4244	0.9979
experiment	1.7973	1.2859	0.8156			

**Table 6:** Combination of simulated annealing (SA) parameter rates.

Parameters	Setting Value/Function Type
Objective limit	1 × 10^−4^
Annealing function	Boltzmann annealing
Reannealing interval	100
Temperature update function	Exponential temperature
Initial temperature	100
Acceptance probability function	Simulated annealing acceptance
Data type	Double
